# Diet and Nutritional Status of Women of Reproductive Age (15–49 Years) in Indigenous Communities of *Attappady*, Kerala, India

**DOI:** 10.3390/nu16162698

**Published:** 2024-08-14

**Authors:** P. V. Sunu, Abdul Jaleel, G. Neeraja, G. Jayalakshmi, D. Narasimhulu, B. Senthilkumar, T. Santhoshkumar, K. Sreeramakrishna, N. Arlappa

**Affiliations:** 1 Division of Public Health Nutrition, ICMR-National Institute of Nutrition (NIN), Hyderabad 500007, India; sunupv1@gmail.com (P.V.S.); daraneeraja14@gmail.com (G.N.); drjayalakshmi969@gmail.com (G.J.);; 2 Department of Epidemiology Statistics, ICMR-National Institute for Research in Tuberculosis (NIRT), Chennai 600031, India

**Keywords:** minimum dietary diversity, micronutrient intake, chronic energy deficiency, anemia, *Attappady*

## Abstract

The dietary patterns and quality of diets of women of reproductive age (WRA) significantly affect their health and that of their children. The suboptimal diet among women can lead to issues such as intrauterine growth retardation, low birth weight, premature birth, and malnutrition. To examine the dietary patterns and nutrient intake of WRA in the indigenous communities of the *Attappady* tribal block in Kerala, we conducted a cross-sectional study in 20 randomly selected villages in 2022. The study involved 24 h dietary recall surveys, anthropometric measurements, and estimation of hemoglobin concentration to assess nutrient intake and nutritional status. A total of 446 women aged 15–49 from 423 households participated, with 92 households included in the diet survey. The findings indicated that the diet was primarily based on cereals and root-based starchy staples, with low consumption of dairy products, fruits, and vegetables. The estimated intakes of major nutrients, except for protein, were lower than the recommended dietary allowance (RDA). Nearly 50% of the WRA were malnourished. About 32% of non-pregnant and non-lactating (NPNL) women and 40% of lactating mothers suffered from chronic energy deficiency (BMI < 18.5 kg/m^2^). Conversely, 13.4% of NPNL women and 15% of lactating mothers were overweight or obese (BMI ≥ 25 kg/m^2^). A total of 12.5% of adolescent girls aged 15–19 were thin (BAZ < −2 SD), and 10.5% were overweight or obese (BAZ > +1 SD). Since the co-existence of micronutrient deficiencies and undernutrition is rooted in the socio-cultural aspects of indigenous tribes, a culturally sensitive nutrition intervention model would be appropriate for the better health and wellbeing of women in the community.

## 1. Introduction

The United Nations *Permanent Forum on Indigenous Issues* defines Indigenous Peoples as those maintaining historical continuity with a specific region before colonization or annexation [1]. These communities maintain strong ties to their territories, natural resources, and ecosystems, preserving distinct social, economic, and political systems, as well as unique languages, cultures, beliefs, and knowledge systems, and access to “traditional foods” through either natural environment farming or wild harvesting [1]. However, the impact of colonization in various parts of the world has resulted in the subjugation and dispossession of Indigenous Peoples, leading to a disassociation from their traditional land, culture, linguistic heritage, foods, and identity [2]. This has substantially contributed to disparities in food security, health, and overall well-being [1,3,4]. Indigenous populations are the ones who experience widespread and severe cases of malnutrition across the globe [5,6].

India has the world’s largest tribal population, with 104.3 million people identified as part of the Scheduled Tribe (ST) community, comprising 8.6% of the country’s total population [7]. Recent data underscore the enduring challenges of undernutrition, revealing that 35.5% of children aged 0–59 months suffer from stunted growth, 19.3% are affected by wasting, and 32.1% are underweight [8]. Studies in Maharashtra, Jharkhand, Madhya Pradesh, and Kerala further highlighted disturbing levels of undernutrition, including high rates of stunting (40 to 60%), wasting (20 to 27%), and underweight (48 to 53%) among tribal children aged 0–59 months [9,10,11,12]. Additionally, there is a concerning prevalence of anemia (91.2%) and deficiencies in iron (50%), and vitamin B12 (35%), among children aged 12–59 months [9].

This study was carried out in *Attappady*, renowned as a tribal heartland in Kerala, India. The state of Kerala is known for its advancements in the health sector and is appreciated for its impressive achievements in social sectors comparable to those of many developing countries [13]. However, tribal populations in the state, notably in the *Attappady* region, inhabited by indigenous tribes such as the *Irula*, *Muduga*, and *Kurumba* communities, stand out for their high levels of malnutrition and neonatal and infant mortality. Notably, the region has witnessed 136 infant deaths from 2012 to 2022, primarily attributed to factors such as premature delivery, low birth weight, pre-eclampsia, acute respiratory distress syndrome (ARDS), and anemia, with a substantial number of mothers reporting nutritional deficiencies [14].

It is known that women of reproductive age (WRA) have distinct nutritional needs, particularly before and during pregnancy and while breastfeeding. They require nutritious diets to build sufficient reserves for pregnancy and ensure the survival and well-being of both mothers and their children. A woman’s diet lacking key nutrients such as iodine, iron, folate, calcium, and zinc can lead to stillbirth, low birth weight, wasting, and developmental delays in children [15]. Additionally, deficiencies in these nutrients can cause anemia, pre-eclampsia, hemorrhage, and maternal death [15,16,17]. Hence, poor maternal diet and nutrition are recognized as significant risk factors for adverse maternal and child health outcomes.

Improving the overall health and wellbeing of indigenous tribes in *Attappady* requires addressing the nutritional and health needs of women in the community. Understanding the nutritional gap and women’s malnutrition is crucial for this [18]. In the context of the high prevalence of malnutrition and neonatal mortality in *Attappady*, examining the dietary patterns of women is important. To the best of our knowledge, there is no published article addressing the issue of dietary gaps in WRA in *Attappady*. In this paper, we aimed to study the food and dietary intakes and nutritional status of tribal women of reproductive age (15–49 years) in *Attappady*. Further, we discuss the potential consequences of poor nutritional status among women, including its impact on child health and mortality, and its role in perpetuating the intergenerational transmission of malnutrition and poverty within these communities.

## 2. Materials and Methods

The present paper is part of a larger study called the comprehensive nutritional assessment of tribal children aged 0–59 months in *Attappady*. Details about the study and its methods are reported elsewhere [9].

### 2.1. Study Design

This was a cross-sectional study conducted in the *Attappady* block of the Palakkad district, Kerala, India, from May to June 2022.

### 2.2. Study Population

As per the 2011 census, the tribal population in *Attappady* Block was 27,627, consisting of 13,708 men and 13,919 women [7]. For this study, we randomly selected 20 villages from a list of 192 provided by the Integrated Tribal Development Project (ITDP) office in *Attappady*. This study therefore covered a substantial geographical area and included a significant population (446 women from 423 households). The present study represents all tribal women aged 15–49 years within the *Attappady* tribal block.

### 2.3. Sample Size

The primary objective of this study was to assess the nutritional status of children aged 0–59 months. The sample size calculation was performed based on a previous ICMR-National Institute of Nutrition (NIN) assessment, which found a 48.5% prevalence of underweight in this age group [14]. The estimated sample size for the study was 340 children (aged 0–59 months). The sample size was increased to 400 children to account for potential refusals to provide blood samples. In each selected village, we chose 20 households with at least one child aged 0–59 months for data collection. If a village had less than 20 such children, we included an adjacent village to obtain the remaining children. We collected relevant information from all women aged 15–49 present during the visit to assess their diet and nutritional status.

### 2.4. Study Tools

Household socioeconomic data were collected using a structured questionnaire. Anthropometric measurements (height and weight) of all available women in the selected households were measured and recorded using standard instruments and methods. The Seca 813 digital weighing scale and Seca 213 stadiometer (Seca, Hamburg, Germany) were used to measure weight and height, respectively. A diet survey was conducted among 25% of the selected households to gather the dietary information of household members using the one-day, 24 h dietary recall method. This method involved a structured interview to obtain detailed information on all foods and beverages consumed by the household members, including WRA, in the past 24 h, from midnight to midnight of the previous day. The hemoglobin status of WRA was measured using the hemocue method. For this, a finger-prick blood sample of 20 μL was collected and dropped on a Hb strip.

### 2.5. Data Analysis

All statistical analyses were conducted using Stata-14, a software package developed by Stata Corp. (College Station, TX, USA). For the calculation of BMI and anemia, WHO cut-off values were used [19,20]. From the diet data, the daily average consumption of various foods was calculated for individuals based on their age, physiological status, and physical activity level. The content of various nutrients in the foods consumed by women was calculated using the Indian Food Composition Tables (IFCT) [21] and the Nutritive Value of Indian Foods [22]. The median intake of various nutrients was compared against the Recommended Dietary Allowances (RDA) for Indians as recommended by the ICMR [22]. Dietary diversity was calculated using the methodology proposed by the Food and Agriculture Organization (FAO) for calculating Minimum Dietary Diversity for Women (MDD-W) [23]. MDD-W is a dichotomous variable of whether women 15–49 years of age have consumed at least five out of ten defined food groups during the last 24 h.

## 3. Results

### 3.1. Profile of the Study Area and Population

This study included 446 women, aged 15–49, from 423 households. Of these, 308 were Non-Pregnant and Non-Lactating (NPNL), 40 were currently pregnant, and 98 were lactating mothers. Anthropometric data were collected from 445 women, while hemoglobin levels were assessed for 187 of them. A 24 h recall diet survey was conducted in 92 households, consisting of 92 women of reproductive age.

The average household comprised 4.5 members, predominantly from nuclear families (72%). Around 80% of the households belonged to the *Irula* tribe, 13% to the *Muduga* tribe, and 7% to the *Kurumba* tribe. Approximately 45% of households had access to safe drinking water, while 42% relied on streams and other unprotected sources. Firewood was the primary cooking fuel for the majority (69%) of households, and 76% had access to sanitary latrines.

In *Attappady*, three Primary Health Centers (PHCs), including one Community Health Center (CHC), and a Tribal Specialty Hospital, along with a few hospitals supported by Non-Governmental Organizations are providing free healthcare services. Given the high prevalence of undernutrition, the region has three Nutrition Rehabilitation Centers (NRCs). Each tribal hamlet has a tribal promoter to facilitate community access to government services. Additionally, Accredited Social Health Activists (ASHA) and *Anganwadi* Workers (AWW) are providing services to link the community with the healthcare system as well as providing various nutrition services.

In the community, one-fifth of WRA were unable to read or write. The average age at marriage was 19.6 years. One-third of women in this age group consume tobacco on a regular basis. Three out of every four adolescent girls were provided with iron and folic acid (IFA) tablets in the previous year. However, only 50% of them stated that they were actually consuming the IFA tablets. Additionally, only 45% of the adolescent girls received deworming tablets in the past year, and only half of them consumed the tablets. Almost all pregnant women (97%) reported receiving antenatal care (ANC), and 87% of them consumed IFA tablets. Nearly all pregnant women (96%) received benefits from Integrated Child Development Services (ICDS), particularly food supplementation. The majority (98%) of lactating mothers delivered at hospitals. The mean birth weight of infants was 2.9 kg, with 39% of babies born with a low birth weight (<2500 g) and 4% born with a very low birth weight (<1500 g).

### 3.2. Dietary Intake of Women of Reproductive Age in Attappady

The analysis of 24 h diet recall data revealed that nearly all women (99.2%) had consumed two or more major meals within the 24 h preceding the survey. Their typical dietary pattern leaned heavily towards cereals, with parboiled rice emerging as the primary staple and very few (<1%) consuming millets. On average, the intake of most food groups fell below the daily requirement level, except for grains, roots, tubers, and pulses.

**Figure 1:**
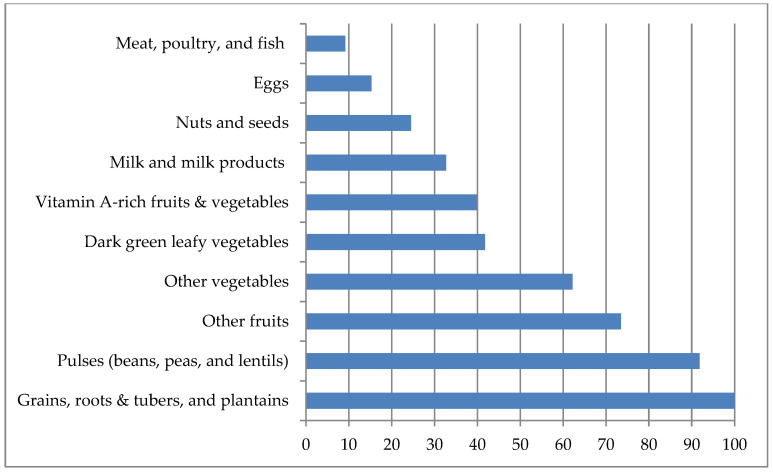
Dietary diversity among women of reproductive age in *Attappady*.

Figure 1 shows the percentage of WRA who consumed each major food group (the 10 food groups required for calculating MDD-W). The most prominent food groups consumed by WRA were ‘grains, roots, and tubers’ (100%), followed by pulses (92%), other fruits (73%), and other vegetables (62%). However, consumption of other food groups such as green leafy vegetables, vitamin A-rich fruits and vegetables, milk, and milk products was notably below 50% of WRA. Minimal intake of nuts and seeds, eggs, and meat was observed among WRA. The study revealed that 65% of women had minimum dietary diversity for women (MDD-W). We have also observed that the availability of many of these food items is seasonal, which can affect dietary diversity, particularly other fruits during lean seasons.

The mean intake of foods consumed by each physiological category of women is shown in Table 1. Among the food groups, roots and tubers have the highest consumption, whereas the consumption of green leafy vegetables and milk and milk products is notably below the daily requirement.

The median intake of major nutrients by WRA in *Attappady* is shown in Table 2. The data are presented based on the physiological status of women. Energy and protein intake were below the RDA for women except for NPNL. The estimated intake of most essential nutrients, including fat, calcium, iron, vitamin A, riboflavin, vitamin C, and zinc, fell below the RDA. The daily intake of calcium was notably low across all physiological groups of women, reaching only 36% of the RDA for NPNL women, 43% for pregnant women, and 24% for lactating mothers. Despite this, WRA in *Attappady* obtained only below 50% of the required iron from their diet. Fat intake was also below the RDA for all three groups, with lactating mothers experiencing the most severe deficit.

Table 3 shows the percentage of women meeting the RDI for nutrients. The majority (80%) of WRA were not meeting at least 50% of the required levels for calcium, iron, and riboflavin. Intake of vitamin A was also very low, with 60% to 80% of them consuming less than 50% of the required amount through their daily diet.

### 3.3. Nutritional Status of Women of Reproductive Age in Attappady

**Figure 2:**
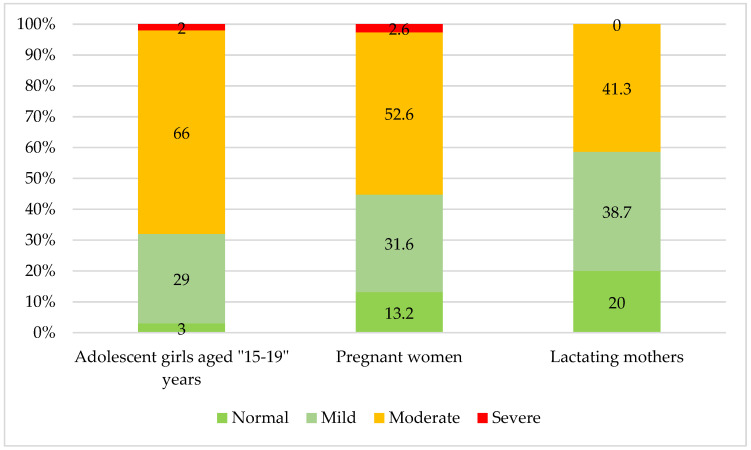
Prevalence of anemia among women of reproductive age in *Attappady*.

Among NPNL women, 32% had chronic energy deficiency (BMI < 18.5 kg/m^2^), while among lactating mothers, this was 40%. On the other hand, overweight and obesity (BMI ≥ 25) were observed in 13% of NPNL women and 16% of lactating mothers. Among adolescent girls aged 15–19, 12.5% were thin (BAZ < −2 SD), and 10.5% were overweight or obese (BAZ > +1 SD). The majority of WRA in *Attappady* suffer from anemia, with prevalence rates of 97% among adolescent girls aged 15–19 years, 87% among pregnant women, and 80% among lactating mothers (Figure 2).

## 4. Discussion

The current study was conducted in a tribal area of Kerala state, which is frequently highlighted in the news due to poverty-related challenges, undernutrition, neonatal, and infant mortality [9,14]. Malnutrition is found to be a major public health concern in the community, as there is a high level of CED, overweight/obesity, and anemia among WRA. Additionally, women are unable to meet the daily requirement for most of the nutrients, leading to the perpetuation of the malnutrition cycle in the community.

This study shows that women’s diets are primarily dominated by grains, roots, and tubers, with pulses and legumes also being common staples. This is resulting in energy intake being nearly adequate compared to the RDA. Despite this, their diet lacks minimal diversity, particularly in green leafy vegetables, vitamin A-rich fruits and vegetables, and dairy products, with over one-third failing to obtain the MDD-W. Key nutrient intake falls well below the RDA, especially for calcium, iron, and vitamin A, which are essential in this reproductive age. In response to these challenges, the government of Kerala has implemented several community supportive programs. Through a targeted public distribution system, the government provides free grains, such as rice and pulses, to households, helping them mitigate food insecurity. Approximately 92% of households benefit from this system [9]. Mothers of children under five years of age have received Take Home Ration (THR) and cooked meals from ICDS Anganwadi Centers. All school-going adolescent girls receive meals through the mid-day meal (MDM) program. Additionally, almost all the tribal hamlets in *Attappady* have community kitchens supported by the government of Kerala. These initiatives aim to meet the energy requirements (800 kcal and 21 g of protein per day) of the most vulnerable members of the community, including pregnant women, lactating mothers, children aged 6 months to 6 years, the elderly, and destitute individuals. Because of the free grain-based food distribution, people in the community are assured of receiving food every day and may not cook anything else, relying solely on this provision. This reliance on food from the community kitchens, which is aimed at meeting energy and protein requirements, is likely to cause severe deficiencies in other nutrients. This paradox emerges from the inclination towards freely accessible food items from the public distribution system, readily available meals from community kitchens, and the extensive availability of low-cost, ready-made foods.

The persistent food and nutrition insecurity experienced by the community has had lasting effects on the health and well-being of its young women and children. The presence of poverty and malnutrition within the community can be traced to several factors, including the change in the agrarian system. This transition is influenced by diverse factors, including climate change, cost constraints, and land scarcity. Moreover, a noteworthy shift has occurred from traditional forest-based livelihoods to food systems towards wage labor.

Monotonous diets, mainly consisting of starchy staples and lacking sufficient vegetables, fruits, and animal-derived foods, contribute to malnutrition among low-income individuals [24]. Our observation in *Attappady* indicates that despite the availability of leafy vegetables and millet at the production level, they are not adequately utilized in consumption. This phenomenon is not unique to the indigenous communities in *Attappady* but is also observed among many other indigenous groups. The gap between availability and consumption is influenced by various factors, including demanding work schedules, evolving cultural preferences towards rice-based staples, transitioning from traditional agriculture to wage labor, and the underutilization of locally diverse food sources [25,26,27].

In *Attappady*, many women have experienced abortions, premature deliveries, and the low birth weight of their children. Inadequate nutrition during the preconception period can lead to intrauterine growth retardation and fetal stunting, increasing the likelihood of an undernourished mother delivering an undernourished baby [28]. The pathophysiology of conditions like pre-eclampsia is closely associated with maternal diet, highlighting the importance of balanced nutrition in preventing such complications and supporting optimal placental and fetal development [29]. Zinc is also crucial for fetal growth and overall neonatal health, with animal-based foods being the primary source; however, the present study indicates a deficiency of zinc ranging from 37% to 46% in the community’s diet, potentially contributing to high rates of premature deliveries and neonatal deaths [30].

Iron deficiency anemia is another significant factor leading to adverse pregnancy outcomes, including preterm birth and poor infant growth [31]. In the community, the primary source of iron from the daily diet is staging due to insufficiency in consumption, whereas the other source from supplementary iron, mostly tablets, is reported to be under consumption as they do not comply with health system norms [32,33]. The reported deaths in the community may be epidemiologically linked to multiple nutrient deficiencies.

The traditional practices of hunting, fishing, and gathering edible forest resources, particularly green leafy vegetables, have significantly diminished within the community over a period of time. Previously, the diet of the tribal people of *Attappady* comprised approximately 60 varieties of green vegetables and millets [34]. This shift away from their ecosystem-based food practices towards governance by non-indigenous systems, including land alienation, has eroded their traditional ways of eating. Additionally, resistance to health norms stems from a historical disconnect from mainstream populations and a fear of negative consequences, leading to a reluctance to adhere to health interventions like iron-folic acid supplements and deworming tablets. To effectively address nutritional challenges, the culturally sensitive implementation of programs tailored to the community’s unique cultural context is required.

This study has some limitations. First, the one-day, 24 h diet recall covered only 22% of the selected households, which include 92 WRA. Therefore, the food and nutrient intake calculations are based on this limited number of women. Another limitation is that a single-point examination of diet can be affected by seasonal factors, such as the availability or non-availability of seasonal fruits, vegetables, and other forest resources.

## 5. Conclusions

Despite the presence of healthcare facilities, nutritional rehabilitation centers, community kitchens, and cash benefit transfers, the tribal population in *Attappady* continues to struggle with a high prevalence of undernutrition. The root of this problem is complex and extends beyond the mere lack of essential services. To address nutrient inadequacy through diet, it is crucial to develop and implement a culturally sensitive nutrition intervention model aimed at enhancing the nutrition knowledge of women and augmenting the production and consumption of nutrient-rich fruits and vegetables. This model should be co-developed and led by the community to revive their traditional food systems that enable healthy food choices throughout the year.

## Figures and Tables

**Table 1:** Mean intake of food stuffs (g/day) among women of reproductive age in *Attappady*.

Food Groups	NPNL-Sedentary (*n* = 31)	NPNL-Moderate (*n* = 34)	Pregnant Women (*n* = 5)	Lactating Mothers (*n* = 23)
	Mean (±SD) (in Grams)	Mean (±SD) (in Grams)	Mean (±SD) (in Grams)	Mean (±SD) (in Grams)
Cereals and Millets	323.0 (121)	357.4 (112)	370.8 (147)	366.2 (142)
Pluses and Legumes	61.6 (40)	66.5 (36)	94.0 (38)	77.0 (38)
Green Leafy Vegetables	20.8 (33)	22.9 (31)	53.4 (40)	21.3 (29)
Other Vegetables	56.4 (85)	47.5 (57)	34.1 (56)	71.2 (70)
Roots and Tubers	98.2 (73)	116.3 (88)	86.5 (39)	113.6 (59)
Nuts and Oils seeds	8.6 (15)	9.7 (15)	7.0 (6)	11.8 (23)
Condiments and Spices	16.5 (12)	17.5 (11)	21.7 (7)	18.1 (9)
Fruits	41.0 (43)	47.5 (43)	50.0 (34)	37.2 (31)
Other Flesh Foods	18.7 (29)	7.1 (20)	23.8 (53)	16.7 (30)
Milk and Milk Products	70.0 (109)	30.8 (55)	40.1 (54)	44.6 (78)
Fats and Oils	13.2 (8)	11.1 (6)	20.7 (13)	10.8 (5)
Sugar and Jaggery	27.0 (11)	28.5 (15)	33.7 (23)	22.4 (10)

SD: Standard Deviation; NPNL: Non-pregnant non lactating.

**Table 2:** Median intake of nutrients among women of reproductive age in *Attappady*.

Nutrients	NPNL (*n* = 65)		Pregnant Women (*n* = 5)		Lactating Mothers (*n* = 23)	
	Median (±SD)	RDA	Median (±SD)	RDA	Median (±SD)	RDA
Energy (kcal)	1873.5 (505)	1660.0	1949.9 (564)	2010.0	1945.9 (526)	2220.0
Protein (g)	53.5 (12)	46.0	63.6 (3)	68.0	57.6 (13)	61.0
Total Fat (g)	21.9 (17)	28.0	39.4 (15)	46.0	21.9 (13)	49.0
Calcium (mg)	287.8 (283)	800.0	426.4 (190)	1000.0	279.9 (136)	1200.0
Iron (mg)	8.7 (6)	15.0	14.8 (7)	27.0	11.3 (3)	23.0
Vitamin A (µg)	185.5 (356)	840.0	60.5 (446)	900.0	359.0 (495)	950.0
Thiamin (mg)	1.0 (0.3)	1.1	1.6 (0.2)	2.0	1.3 (0.3)	2.1
Riboflavin (mg)	0.7 (0.2)	1.6	0.7 (0.1)	2.7	0.7 (0.2)	2.9
Niacin (mg)	11.4 (3)	9.0	12.7 (3)	13.5	12.3 (3.5)	16.0
Vitamin C (mg)	40.8 (33)	55.0	83.9 (45)	70.0	48.5 (37.4)	115.0
Total Folate (µg)	230.2 (548)	180.0	279.2 (61)	570.0	254.4 (78.6)	330.0
Zinc (mg)	7.1 (2)	11.0	9.2 (0.8)	14.5	7.9 (2.3)	14.1

SD: Standard Deviation; RDA: Recommended Dietary Allowance.

**Table 3:** Percentage of women meeting RDA as per nutrient intake (per day).

Nutrient	Physiological Category	<50% of RDA	50–70% of RDA	≥70% of RDA
Protein (g)	NPNL-Sedentary	3.2	-	96.8
	NPNL-Moderate	-	-	100
	Lactating Mother	4.3	-	95.7
Fat (g)	NPNL-Sedentary	19.4	22.6	58.1
	NPNL-Moderate	14.7	26.5	58.8
	Lactating Mother	17.4	21.7	60.9
Energy (kcal)	NPNL-Sedentary	-	9.7	90.3
	NPNL-Moderate	-	5.9	94.1
	Lactating Mother	4.3	-	95.7
Calcium (mg)	NPNL-Sedentary	87.1	6.5	6.5
	NPNL-Moderate	88.2	8.8	2.9
	Lactating Mother	91.3	4.3	4.3
Iron (mg)	NPNL-Sedentary	93.5	-	6.5
	NPNL-Moderate	91.2	8.8	-
	Lactating Mother	82.6	17.4	-
Vitamin A (µg)	NPNL-Sedentary	80.6	-	19.4
	NPNL-Moderate	64.7	14.7	20.6
	Lactating Mother	60.9	8.7	30.4
Thiamine (mg)	NPNL-Sedentary	9.7	38.7	51.6
	NPNL-Moderate	2.9	32.4	64.7
	Lactating Mother	4.3	17.4	78.3
Riboflavin (mg)	NPNL-Sedentary	87.1	-	12.9
	NPNL-Moderate	91.2	-	8.8
	Lactating Mother	87.0	-	13
Niacin (mg)	NPNL-Sedentary	-	16.1	83.9
	NPNL-Moderate	2.9	2.9	94.1
	Lactating Mother	4.3	4.3	91.3
Vitamin C (mg)	NPNL-Sedentary	38.7	12.9	48.4
	NPNL-Moderate	35.3	23.5	41.2
	Lactating Mother	21.7	26.1	52.2
Dietary Folate (µg)	NPNL-Sedentary	6.5	12.9	80.6
	NPNL-Moderate	8.8	8.8	82.4
	Lactating Mother	4.3	-	95.7
Zinc (mg)	NPNL-Sedentary	38.7	54.8	6.5
	NPNL-Moderate	38.2	47.1	14.7
	Lactating Mother	21.7	56.5	21.7

RDA: Recommended Dietary Allowance; NPNL: Non-pregnant non lactating.

## Data Availability

The data will be available as per the request. Data not publicly available due to legal reasons.

## References

[1] Myrna C. (2009). The State of the World’s Indigenous Peoples.

[2] Malli A., Monteith H., Hiscock E.C., Smith E.V., Fairman K., Galloway T., Mashford-Pringle A. (2023). Impacts of colonization on Indigenous food systems in Canada and the United States: A scoping review. BMC Public Health.

[3] King M., Smith A., Gracey M. (2009). Indigenous health part 2: The underlying causes of the health gap. Lancet.

[4] Smallwood R., Woods C., Power T., Usher K. (2021). Understanding the impact of historical trauma due to colonization on the health and well-being of indigenous young peoples: A systematic scoping review. J. Transcult. Nurs..

[5] Anderson I., Robson B., Connolly M., Al-Yaman F., Bjertness E., King A., Tynan M., Madden R., Bang A., Coimbra C.E. (2016). Indigenous and tribal peoples’ health (The Lancet-Lowitja Institute Global Collaboration): A population study. Lancet.

[6] Rivadeneira M.F., Moncayo A.L., Cóndor J.D., Tello B., Buitrón J., Astudillo F., Caicedo-Gallardo J.D., Estrella-Proaño A., Naranjo-Estrella A., Torres A.L. (2022). High prevalence of chronic malnutrition in indigenous children under 5 years of age in Chimborazo-Ecuador: Multicausal analysis of its determinants. BMC Public Health.

[7] ORGI (2011). Census of India—SCHEDULED TRIBES.

[8] IIPS (2021). National Family Health Survey-5.

[9] Jaleel A., Arlappa N., Ramakrishna K.S., Sunu P.V., Jayalakshmi G., Neeraja G., Narasimhulu D., Kumar T.S., Kumar S.B. (2023). Examining the Triple Burden of Malnutrition: Insights from a Community-Based Comprehensive Nutrition Survey among Indigenous Tribal Children (0–19 Years) in the Western Ghats Hills of India. Nutrients.

[10] Ghosh S., Varerkar S.A. (2019). Undernutrition among tribal children in Palghar district, Maharashtra, India. PLoS ONE.

[11] Ghosh-Jerath S., Singh A., Bhattacharya A., Ray S., Yunus S., Zodpey S.P. (2013). Dimensions of nutritional vulnerability: Assessment of women and children in Sahariya tribal community of Madhya Pradesh in India. Indian J. Public Health.

[12] Ghosh-Jerath S., Kapoor R., Singh A., Nilima, Downs S., Goldberg G., Fanzo J. (2021). Agroforestry diversity, indigenous food consumption and nutritional outcomes in Sauria Paharia tribal women of Jharkhand, India. Matern. Child Nutr..

[13] Kutty V.R. (2000). Historical analysis of the development of health care facilities in Kerala State, India. Health Policy Plan..

[14] NIN (2013). A Rapid Assessment of Nutritional Status of Underfive Year Children & Mothers of Attappadyhills, Palakkad District of Kerala and Cause of Infant Deaths by Verbal Autopsy.

[15] Ramakrishnan U. (2004). Nutrition and low birth weight: From research to practice. Am. J. Clin. Nutr..

[16] Farias P.M., Marcelino G., Santana L.F., de Almeida E.B., Guimarães R.C.A., Pott A., Hiane P.A., Freitas K.C. (2020). Minerals in Pregnancy and Their Impact on Child Growth and Development. Molecules.

[17] Walle B.M., Adekunle A.O., Arowojolu A.O., Dugul T.T., Mebiratie A.L. (2022). Low birth weight and its associated factors in East Gojjam Zone, Amhara, Ethiopia. BMC Nutr..

[18] Thomas S.T., Thomas E.T., McLean M., Titus T.T. (2021). Paving the way to achieving the United Nations Sustainable Development Goals for women from Indigenous communities: Lessons from Attappady, India. Discov. Sustain..

[19] WHO (2011). Haemoglobin Concentrations for the Diagnosis of Anaemia and Assessment of Severity.

[20] WHO (2000). Obesity: Preventing Managing the Global Epidemic: Technical Report Series 894.

[21] Longvah T., Ananthan R., Bhaskarachary K., Venkaiah K. (2017). Indian Food Composition Tables.

[22] ICMR (2020). Report of the Expert Group of the ICMR: Nutrient Requirements and Recommended Dietary Allowances for Indians.

[23] FAO (2021). Minimum Dietary Diversity for Women.

[24] Black R.E., Victora C.G., Walker S.P., Bhutta Z.A., Christian P., de Onis M., Ezzati M., Grantham-McGregor S., Katz J., Martorell R. (2013). Maternal and child undernutrition and overweight in low-income and middle-income countries. Lancet.

[25] Ghosh-Jerath S., Kapoor R., Bandhu A., Singh A., Downs S., Fanzo J. (2022). Indigenous Foods to Address Malnutrition: An Inquiry into the Diets and Nutritional Status of Women in the Indigenous Community of Munda Tribes of Jharkhand, India. Curr. Dev. Nutr..

[26] Kuhnlein H., Erasmus B., Spigelski D. (2009). Indigenous Peoples’ Food Systems: The Many Dimensions of Culture, Diversity and Environment for Nutrition and Health.

[27] McCune L.M., Nuvayestewa V., Kuhnlein H.V. (2019). Why and How to Document the Traditional Food System in your Community: Report from Breakout Discussions at the 2017 Native American Nutrition Conference. Curr. Dev. Nutr..

[28] Setiawan A.S., Indriyanti R., Suryanti N., Rahayuwati L., Juniarti N. (2022). Neonatal stunting and early childhood caries: A mini-review. Front. Pediatr..

[29] Kinshella M.W., Omar S., Scherbinsky K., Vidler M., Magee L.A., von Dadelszen P., Moore S.E., Elango R. (2022). Maternal nutritional risk factors for pre-eclampsia incidence: Findings from a narrative scoping review. Reprod. Health.

[30] Shah D., Sachdev H.P. (2006). Zinc deficiency in pregnancy and fetal outcome. Nutr. Rev..

[31] Allen L.H. (2000). Anemia and iron deficiency: Effects on pregnancy outcome. Am. J. Clin. Nutr..

[32] George M.S., Davey R., Mohanty I., Upton P. (2020). “Everything is provided free, but they are still hesitant to access healthcare services”: Why does the indigenous community in Attapadi, Kerala continue to experience poor access to healthcare?. Int. J. Equity Health.

[33] Haddad S., Mohindra K.S., Siekmans K., Màk G., Narayana D. (2012). “Health divide” between indigenous and non-indigenous populations in Kerala, India: Population based study. BMC Public Health.

[34] Panikker M.J. Altering Perspectives and Preserving Diversities: A Look into Kerala’s Tribal Reform. Proceedings of the Third 21st CAF Conference at Harvard.

